# Funding Graduate Programs in Gerontology in a Time of Fiscal Constraint

**DOI:** 10.1093/geroni/igaf122.1038

**Published:** 2025-12-31

**Authors:** Debra Dobbs, William Haley

**Affiliations:** University of South Florida, Tampa, Florida, United States; University of South Florida, University of South Florida, Florida, United States

## Abstract

Graduate research programs in the United States are funded in a myriad of ways and differ based on if they are part of a public versus private institution. Such programs can face a variety of pressures, including cuts in state or federal funding, varying levels of research grant funding on certain topics, new priorities for research funding, state funding that is dependent on productivity goals (e.g., four-year undergraduate graduation rates or major headcounts). Proposed reductions in indirect cost rates for grants from the National Institute on Aging and the National Science Foundation are a new potential challenge in funding doctoral students in aging research programs. This paper presents strategies to sustain graduate student research programs through a time of federal and state economic uncertainty based on experiences with an Aging Studies graduate program from a large (55K+ student population) Florida public university that is part of the Association of American Universities (AAU) with several research doctoral programs. Strategies that have been used in the past, or that are being used currently include admitting some students part-time without financial support, increased funding of students through teaching assistantships, efforts to enhance funding from private sources such as foundations and donors and admitting smaller cohorts of students. Discussion with the panelists and the audience will include views on these and other approaches to sustaining graduate student research programs in aging given the uncertain nature of the political and economic climate.

